# Relief Demand Calculation in Humanitarian Logistics Using Material Classification

**DOI:** 10.3390/ijerph17020582

**Published:** 2020-01-16

**Authors:** Jianfang Shao, Changyong Liang, Xihui Wang, Xiang Wang, Liang Liang

**Affiliations:** 1 School of Management, Hefei University of Technology, Hefei 230000, China; cyliang@hfut.edu.cn (C.L.); lliang@hfut.edu.cn (L.L.); 2 School of Management, University of Science and Technology of China, Hefei 230000, China; wxihui@ustc.edu.cn (X.W.); xiawa@mail.ustc.edu.cn (X.W.)

**Keywords:** relief supply, supply classification, material demand calculation, disaster management

## Abstract

Demand calculation, which is the base of most logistics decisions and activities, is a critical work in humanitarian logistics (HL). However, previous studies on demand calculation in HL mainly focus on demand forecasting methodology, with many neglecting the checklist of critical supplies and practice background. This work proposes a new method for relief demand calculation by dividing the process into two parts: supply classification and demand calculation. A general method for classifying relief supplies and clarifying the checklist of relief items for multi-disaster and multiple natural scenarios is given in detail, followed by the procedure of demand calculation for each relief material. The authors present a case study to validate the feasibility and effectiveness of the proposed method based on the disaster response practice in China. Detailed lists of relief demand for different types and severities of disaster are provided.

## 1. Introduction

China is one of the most disaster-hit countries worldwide. Reports provided by the United Nations Office for Disaster Risk Reduction indicated that China experienced direct economic losses valued at 492.2 billion USD between 1998 and 2017, second only to the United States [1]. In recent years, disaster management has attracted considerable attention from the Chinese government, and it was explicitly mentioned in the 13th Five-year Plan of China [2]. In contrast to domestic research attention, the results of China’s disaster management research are unfamiliar to the international academic community [3]. Given the huge disaster burden and disaster relief experience available in China, relief context and operations deserve more attention [3]. Disaster relief operations are inextricably linked to the different departments functioning in the central government of China. For example, the Ministry of Water Resources is responsible for flood control reserves and drought relief supplies, while the Ministry of Civil Affairs is responsible for relief supplies to sustain the daily lives of victims.

Numerous scholars and workers agree that relief supplies are highly important elements of disaster management. Responding to a disaster requires a series of decision-making activities, including demand (type and amount) estimation [4,5], location of temporary distribution facilities [6], allocation and distribution of supplies [7,8], and transportation planning [9,10]. Determining the type of supply demand and calculating the amount of demand for each item for a disaster are critical and foremost for the overall design and implementation of humanitarian logistics (HL) [11,12]. The types of relief items should be determined before relief demand is forecasted, with the analysis of the types of supply demand contributing to reserve and pre-positioning [13]. Studies on inventory, location, distribution, and transportation of relief supplies have attracted a large number of scholars [14,15]. However, few studies have focused on the classification of relief materials or demand calculation to date. No studies have focused on clarifying the checklist of supply demand types and calculating the demand quantity of each relief item.

Recent studies on the material classification problem have mentioned different classification methods according to the urgency and purpose. Different methods have resulted in an unequal number of classes. For example, supplies can be divided into fifteen, five, or eight classes [16,17,18]. However, academia has yet to reach unified classification criteria and methods. Prior studies were unable to clarify the names of the relief items in each category. Consequently, gaining support for decision-making in a real disaster scenario is difficult. Several methods, such as case-based reasoning (CBR) [19,20], grey model [21], fuzzy rough set approach [22], and new hybrid evolutionary-based radial basis function (RBF) networks [23], are used for demand calculation. Decision-makers sometimes use habit or experience to estimate the demand in certain areas [24]. A universal method of calculating these demands in HL is lacking. The conceptual models contributed by these studies are impractical. Hybrid evolutionary-based RBF networks for forecasting supply demand time series is unbeneficial for early demand estimation because it requires long-term accurate early demand as a training set to predict later demand. Membership degrees and characteristic factors are based on subjective experience. The numerical example composed of only a few cases cannot effectively prove the effectiveness of a method. Existing studies did not incorporate material catalogues into a computational approach to generate a practical calculation process. The demand forecasting methods discussed in these studies only considered the forecast of the demand quantity for one or several supplies and ignored the discussion of the types of supply demand. Supply types of demand are an essential part of material demand decision-making [4,25]. Decision-makers must ensure the types of supplies needed for disaster relief and then decide on the demand quantity [26]. However, no research can aid decision-makers on the supplies needed for disaster relief. Therefore, incorporating a material catalogue into a computational approach can make this process practical.

The category and quantity of relief supplies needed during a disaster are essential prerequisites in devising reasonable HL plans. Relief material classification and demand calculation are fundamental points for the follow-up of HL, such as procurement, inventory, transportation, and distribution. A consensus on the concept and composition of demand for disaster relief has yet to be reached. Relief demand depends on multiple factors and varies according to disaster type, season, and region [27,28,29]. Therefore, the following question emerges before an accurate demand calculation is obtained: What constitutes demand? A procedure should be classified prior to the calculation process on the basis of the influencing factors, such as disaster types and natural environment [30,31,32]. The present study proposes a new method for relief demand calculation using material classification. The method consists of two parts: material classification and demand calculation. We set up principles for selecting supplies in the first part. On the basis of these principles, we obtain a collection of relief supplies from various data sources and then classify these supplies according to the type of disasters and natural factors. Finally, we create a material directory that considers the disaster environment and conditions. The latter part focuses on demand calculation. In this part, material demand is calculated after the “disaster impact” and socioeconomic factors are considered. Finally, we apply our ideas and methods to an actual disaster scenario in China and generate quantity lists for the supplies required according to the research framework proposed in this study.

In comparison with previous work on demand calculation of relief supplies, this study offers the following contributions to the HL literature. (1) Material classification and demand calculation are simultaneously considered. Combining the two aspects is a precondition for a reasonable HL plan. (2) A general rule is proposed for material classification and demand calculation in HL. (3) A material catalogue that can be used in Chinese HL practice is developed in accordance with the general rule to determine the demand level by integrating multiresource data.

However, certain questions beyond the scope of this study are worthy of further exploration. First, only few relief supplies are included in this study. The authors excluded from consideration certain relief supplies with a small demand amount to facilitate the study. However, this approach implies that these supplies are needed in disaster relief but only to a certain extent. Second, the discussion on healthcare supplies is a complex question which cannot be solved by an article. No studies have discussed the demand contents of medical supplies and the demand quantity of each medical supply. This work aims to clarify the checklist of demand for medical supplies and provide a rough estimation of demand quantity. Medical supply demand is affected by complex factors, such as number of injured [33], injury severity level [34], and disease progression [35]. A discussion of this complex issue is beyond the length and content of the present work and will be a future research topic. The precise demand or dosage of each drug needs to be determined by experts or clinicians according to the patient’s condition regardless of whether the result of demand estimation is good or not because of the well-known particularity of medical supplies.

The remainder of this study is organized as follows. Section 2 discusses relevant literature on material classification and relief demand calculation. Section 3 describes the proposed classification-based calculation method of relief supplies. Section 4 establishes the proposed models for demand calculation on the basis of material classification. Section 5 describes the proposed classification and calculation processes. Section 6 presents a case study based on a real disaster recorded in China through which the feasibility and effectiveness of the method are validated. Section 5 formulates a numerical case.

## 2. Literature Review

Numerous studies have investigated relief demand, which includes demand types and quantities [5,36,37]. Demand types are obtained by material classification, while demand quantities are obtained by demand calculation. The combination of material classification and demand calculation can help identify the material items reserved in advance and the demand quantities for each item to facilitate rapid response. Although several studies have focused on material classification, they either only briefly mention demand calculation or disregard it, while other studies focus only on the methods of demand calculation and disregard the material items obtained by material classification. Thus far, no studies have focused on clarifying the checklist of supply demand types and calculating the demand quantity of each relief item. In this section, the review of related literature focuses on three aspects, namely material classification, demand calculation, and the combination of material classification and demand calculation.

### 2.1. Relief Material Classification

Pre-storing and pre-positioning critical supplies can help relief organizations deal with demand uncertainty and accelerate relief response [3]. A clearly organized supply classification is convenient for management and optimizes the storage structure. Many studies have mentioned different categories of supply classification based on certain criteria, such as use, priority, characteristics, or emergency management operation [38,39]. In reference to historical relief needs during the 2008 snow disaster and the 5.12 Wenchuan earthquake in China, Wang et al. [40] divided emergency resources into four categories, namely emergency rescue, infrastructure and services, professional resources, and support resource disposal. Zhang [41] divided emergency supplies into four categories according to priority, namely life salvage, engineering support, construction, and reconstruction supplies. Zhang [42] divided relief supplies into three categories according to the relief phases and into five categories according to the reserve characteristics. She et al. developed a four-category classification method based on the construction of the national emergency response capacity [43]. However, none of these studies mentioned the relief items included in each category. Consequently, decision-makers will have difficulty understanding the demand and reserve supplies in advance on the basis of the research results. The mere mention of supply category name of the relief supplies does not determine the critical supplies that are demanded and reserved.

Some studies have introduced classification methods to classify supplies on the basis of the proposed evaluation indices. Ding et al. [44] and Guo et al. [45] classified relief supplies by using the clustering method. Xia et al. [46] proposed a material classification method based on probabilistic neural networks. Wang [47] classified 206 relief supplies into eight classes using the K-mean particle swarm optimization algorithm. Although these studies have classified a certain amount of material items into several categories, they only supposed the number of supply types needed. The classified supplies have not proven to be critical supplies that should be demanded and reserved. Some studies have neglected the actual administrative reserve system and ignored the reality that different departments store different types of supplies. This situation leads to a delay in response owing to administrative coordination. Furthermore, these studies have provided no contents of the material items.

In addition to the academic community, the government and international relief organizations also work to classify supplies. For example, the Chinese government first divided relief supplies into 13 categories in their *Emergency supplies catalogue* (*2004*). The National Development and Reform Commission of China adjusted the categories to 16 items in 2015 under the *Classified catalogue of key supplies for emergency protection* [48]. In the USA, the Federal Emergency Management Agency divides relief supplies into three and eight categories in *Resource Management* (*IS-703*) and *National Incident Management System* resource typing, respectively [49]. International agencies generally divide humanitarian relief supplies into four categories, namely water, sanitation and hygiene; food and nutrition; shelter, settlement, and non-food items; and health care. The Chinese government issued the *Health emergency team equipment reference catalogue (Trial)* [50] for medical supplies, and the U.S. Department of Health and Human Services issued the *Strategic National Stockpile* for medical supply inventory [51]. Most documents clearly provided a variety of relief item names in addition to the material category. However, studies have shown that the set of demanded commodities is relatively small, that is, around only 150 different items; a relatively small number of commodity types capture a sizable portion of the total demands [52]. Pre-storing and pre-positioning the supplies that account for a large number of the demands will improve the efficiency of relief response [52]. The study gap is access to critical supplies that account for sizable portions of the total demands.

This study developed a material classification method to fill the gap. We obtained the demand checklist for multiple disasters and natural scenarios as well as the supply categories in line with the Chinese reserve system through the proposed material classification method and process. First, the supply classification obtains the checklist of demanded supplies that account for a large number of requests by capturing the influencing factors, helping the decision-makers identify the supply demanded and store them in advance and improving the efficiency of the relief response. Second, the supply classification obtains the supply classification rules and results that conform to the Chinese administrative system of supply reserves. Different administrative departments reserve several types of supplies with specific characteristics. Supplies are classified into several family categories, each belonging to an administrative department. The classification results consistent with the administrative system facilitate the coordination of the entire relief system and speed up relief response.

### 2.2. Relief Demand Calculation

In recent years, researchers have introduced several methods to calculate demand. Liu et al. [20] and Wang et al. [19] used the CBR method to calculate demand. However, the effectiveness of CBR must be tested by large numbers of case conditions. Case selection is critical to these studies because the number of cases in the text is small, and the cases significantly differ. Moreover, the operability of the method has not been discussed. Grey model and fuzzy rough set are also used to solve the demand forecast problem [21,22]. However, related studies only provided conceptual models that are difficult to use in practice. Mohammadi et al. [23] developed a hybrid evolutionary-based RBF network for forecasting supply demand time series. However, this method is ineffective for early demand estimation because it requires long-term accurate early demand as a training set to predict later demand. Liu et al. [20] and Wang et al. [53] proposed the demand prediction model on the basis of case-based reasoning. Sun et al. [22] proposed a fuzzy rough set approach. However, membership degrees and characteristic factors are based on subjective experience. The numerical example composed of only a few cases cannot effectively prove the effectiveness of a method. These methods complicate the decision-maker’s estimation of demand immediately after the disaster. The demand forecasting methods discussed in these studies only discuss the forecast of the demand quantity for one or several supplies and ignore the discussion of the types of supply demand. The supply types of demand are an essential part of material demand decision-making [4,25]. Decision-makers need to first ensure the types of supplies needed for disaster relief and then decide on the demand quantity [26]. However, no research can tell decision-makers the supplies needed for disaster relief. Mohammadi et al. [23] used an actual disaster case to verify the feasibility of the proposed model and algorithm. Liu et al. [20], Wang et al. [53], and Sun et al. [22] used a numerical case of simulation to explain the feasibility of the method, which is inadequate. These models lack feasibility in practice. Many researchers ignored the practicability of their methods in disaster management. A few studies provided an example for a feasibility test. Moreover, existing studies did not combine the computational approach with material catalogues to generate a practical calculation process.

This study intends to develop a classification-based calculation method for relief supplies which incorporates material classification and demand calculation into an integrated approach. Our proposed method is used to investigate a large number of real cases and obtain values from these cases. The approach first develops reasonable classification criteria, a demand checklist of each material category, and calculates the demand for each relief material.

### 2.3. Combination of Relief Material Classification and Demand Calculation

Many scholars agree that demand includes types and quantities [4,36,54]. Discussion of type and quantity of material demand is an integral expression, which is indispensable [5,55,56]. Some studies have mixed discussions regarding the influencing factors of supply demand but did not distinguish the influencing factors of different aspects of demand. However, no studies have determined the checklist of contents of demand on the basis of mixed influencing factors. Prior studies have discussed demand quantities for only several supplies. The current study separately discusses the influencing factors that affect demand type and those that affect the amount of material demand. Material classification based on the influencing factors of demand contents can obtain the checklist of contents of demand. Demand calculation can obtain the demand quantity for each material. While material classification and demand calculation are separately discussed, combining them is a good solution to obtain an integral demand (including type and quantity) and a clear checklist of supplies for decision-making.

## 3. Methodology

### 3.1. Conceptual Framework

Relief demand calculation involves two key questions: (1) What items are in demand? (2) How high are the demands? Establishing a material catalogue that basically covers victims’ requirement in clothing, food, shelter, and medical service in a HL operation is the answer to the first question [57,58]. The second question refers to the total quantity of each material to be delivered to the victims. The aspects of content and quantity of demand are focused on to answer the two questions.

In general, relief demand is dependent on various factors, including disaster type, natural environment, and even socioeconomic environment [59,60]. Some studies pile up the factors affecting the demand, which does not separate the influencing factors on the supply types and demand quantity [53,61,62,63]. This situation is nonconducive to clearly accessing the checklist of demand types for multiple disasters and natural scenarios. Moreover, although these studies mentioned the influencing factors of demand, no studies have classified and clearly defined the influencing factors to study the checklist of supply demanded and demand calculation. Therefore, discussing the influencing factors of demand type and demand quantity separately is a good solution for clarifying the checklist of supplies to reserve in advance.

The factors related to demand type are disaster type, seasonal coefficient, climate, weather, and natural environments [53,61,62,63]. No studies have mentioned the socioeconomic factor affecting the types of supply demanded. The changes in demand content caused by socioeconomic factors are negligible in a relief country or region. The influencing factors on contents of demand are disaster type and natural environmental factors. We also interviewed several humanitarian experts and asked them to give their thoughts on the factors that affect the contents of relief demand. The interview result indicated that the main factors affecting the contents of relief material demand are disaster type, season, weather, climate, and temperature. Although the disaster season and the climate conditions of disaster areas are different and the weather or temperature changes, these are generally called the natural environments. An important thing is the human perception caused by the atmospheric environment after the disaster. We introduced an index, that is, apparent temperature proposed by Steadman [63], as a new measure factor of the type of material demanded. Thereafter, we combined different parameter values to simulate various natural state scenarios suitable for multiple disasters and any disaster occurrence time. No other major and important factors affecting the type of material demand have been approved to date. Therefore, the factors affecting the type of material demand are mainly the types of disasters $T_{j}$ and natural scenarios $N_{l}$ [64].

The factors related to demand quantity are gross domestic product (GDP) factor, disaster scale, affected population size, disaster duration, population density, and magnitude [53,61,62,63]. The influencing factors on quantity of demand are disaster impact and socioeconomic factor. The disaster impact refers to the population in need of relief supplies caused by various complex factors after a disaster. The socioeconomic factor, such as GDP, refers to a coefficient that indicates the living standard of victims. Per capita demands should be similar for citizens in rich and poor countries or regions. However, *The State of Food Security and Nutrition in the World* 2019 report provided by United Nations agencies indicated that more than 820 million people or 10.8% of people in the world today are still hungry, and a child dies every 3.6 s because of hunger [65]. Adjusting the per capita distribution is an appropriate choice to reduce deaths due to lack of supplies. Therefore, in this study, we introduced disaster impact $R_{s}$ as the number of victims in need of relief supplies and socioeconomic factor $E_{c}$ as a correlation index to regulate the total demand based on the living standards of victims.

This work is the first to study the checklist of critical supplies demanded for multiple disasters and natural scenarios. A material catalogue ${\left(M_{TN}\right)}_{i}$ with the checklist of critical supply items is obtained by analyzing the disaster types and natural scenarios. The aggregate quantity required for each material ${\left(M_{TN}\right)}_{i}$ is obtained by analyzing the disaster impact and the socioeconomic factors combined with the per capita demand from external standards. This study simultaneously considers material classification and demand calculation for multiple disasters and natural scenarios. Such approach is conducive to identifying the critical supply items with demand quantity and accelerating decision-making on total supply demand, including the types and quantities for relief response.

**Figure 1:**
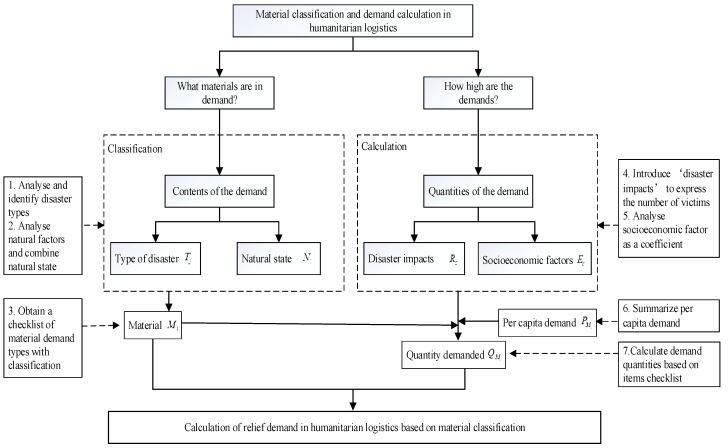
Method of relief demand calculation based on material classification.

Figure 1 presents the classification-based calculation method for relief demand. As shown in Figure 1, the calculation method comprises the following steps:

1. Analyze and identify the type of disaster $T_{j}$ that needs responding to.
2. Analyze the natural factors and their influence on the classification of supplies and combine the natural scenarios $N_{l}$.
3. Obtain a material checklist for disaster relief with material classification.
4. Introduce an indicator called “disaster impact” $R_{s}$ to express the number of victims who need relief supplies.
5. Analyze socioeconomic factor $E_{c}$ and adjust the demand to the living standard of the disaster-affected area.
6. Summarize the per capita needs of international humanitarian aid standards.
7. Calculate demand quantities on the basis of the material checklist and the per capita demand of each type of material.

### 3.2. Computational Model

**Figure 2:**
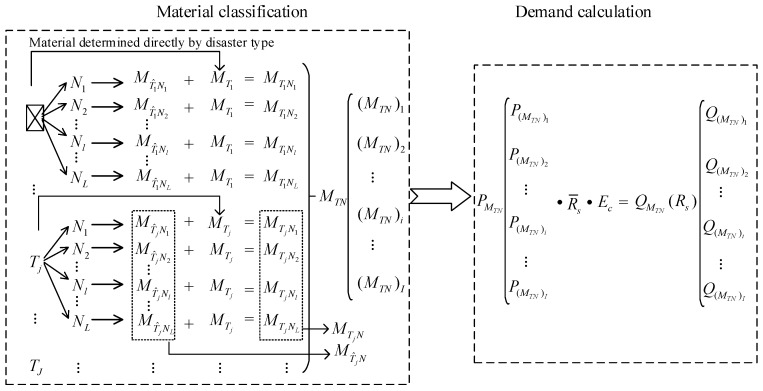
Decision sequence diagram.

The decision-making process is illustrated in Figure 2 for clear and further understanding. Table 1 provides a definition of the symbols to further understand the contents of the decision sequence diagram.

Figure 2 illustrates the decision-making process, wherein relief supplies are divided into two classes. The first one consists of supplies directly determined by disaster type and those determined only after considering natural factors. The second one refers to supplies determined by the natural factors, which is a small but important class.

First, we fix disaster type $T_{j}$ for supplies directly determined by disaster type, which can determine most supplies. For example, we identify the material set $M_{T_{1}}$ by disaster type $T_{1}$. Material set $M_{T_{j}}$ can be determined given a certain disaster type $T_{j}$. Second, we select natural state/conditions $N_{l}$. Certain supplies must be determined by natural factors. Several types of natural factors exist. Various natural states/conditions are obtained by combining several natural factors. A natural state/condition set is expressed as $\left\{N_{1},\cdots ,N_{l},\cdots ,N_{L}\right\}$. A specific natural state $N_{l}$ that corresponds to the existing external environment in terms of major decisions should be established. In disaster type $T_{j}$, material set $M_{{\hat{T}}_{j}N_{l}}$ is required in natural states/conditions $N_{l}$, which are selected within the range of supplies affected by natural environmental factors. For example, in disaster type $T_{1}$ and when natural state/condition is $N_{1}$, the material to be selected is $M_{{\hat{T}}_{1}N_{1}}$, which is chosen within the range of supplies affected by natural environmental factors. Accordingly, the required supplies are $M_{T_{j}N_{l}}$, $M_{T_{j}N_{l}}=M_{T_{j}}+M_{{\hat{T}}_{j}N_{l}}$ for disaster type $T_{j}$ under certain natural states/conditions $N_{l}$. Specifically, the needed supplies are obtained/established by identifying disaster type $T_{j}$ and natural state $N_{l}$. In disaster type $T_{j}$, all supplies affected by natural environmental factors are $M_{{\hat{T}}_{j}N}$, $M_{{\hat{T}}_{j}N}=M_{{\hat{T}}_{j}N_{1}}\cup \cdots \cup M_{{\hat{T}}_{j}N_{l}}\cup \cdots \cup M_{{\hat{T}}_{j}N_{L}}$. Moreover, in a disaster type $T_{j}$, all supplies required are $M_{T_{j}N}$, $M_{T_{j}N}=M_{T_{j}N_{1}}\cup \cdots \cup M_{T_{j}N_{l}}\cup \cdots \cup M_{T_{j}N_{L}}$. Many supplies apply to various types of disasters. For example, material tents are suitable for earthquakes, floods, typhoons, and other disasters. Many supplies needed in different disasters are the same. Several authors are becoming increasingly inclined towards the homogenization of these supplies to facilitate management, inventory, and decision-making. Therefore, the material category “universal set” is a union of supplies needed under various disasters rather than “+” operation. The supplies required for all types of disasters studied in this study are a universal set $M_{TN}$, $M_{TN}=M_{T_{1}N}\cup \cdots \cup M_{T_{j}N}\cup \cdots \cup M_{T_{J}N}$. The material category “universal set” $M_{TN}$ can be expressed as follows: $M_{TN}=({\left(M_{TN}\right)}_{1},{\left(M_{TN}\right)}_{2},\cdots ,{\left(M_{TN}\right)}_{i},\cdots {\left(M_{TN}\right)}_{I})$.

Demand calculation is concise once the classification of supplies is defined. After the supply universal set $M_{TN}$ is determined, per capita demand $P_{{\left(M_{TN}\right)}_{i}}$ of each material ${\left(M_{TN}\right)}_{i}$ can be calculated by referring to international standards [66,67]. The demand quantity $Q_{{\left(M_{TN}\right)}_{i}}$ of each material ${\left(M_{TN}\right)}_{i}$ can be determined by recourse to the disaster impact and a socioeconomic coefficient. We can obtain material set $M_{T_{j}N_{l}}$ and demand quantity set $Q_{M_{T_{j}N_{l}}}$ for dealing with a disaster of type $T_{j}$ and natural state $N_{l}$ by using these steps.

### 3.3. Breakdown of Steps

#### 3.3.1. Classification Process

Material classification is a prerequisite for demand calculation. Classification is used to solve the “contents of demand” problem. The following question is raised: What is “contents of demand”? This section discusses the material catalogues through the classification method to answer this question.

**Figure 3:**
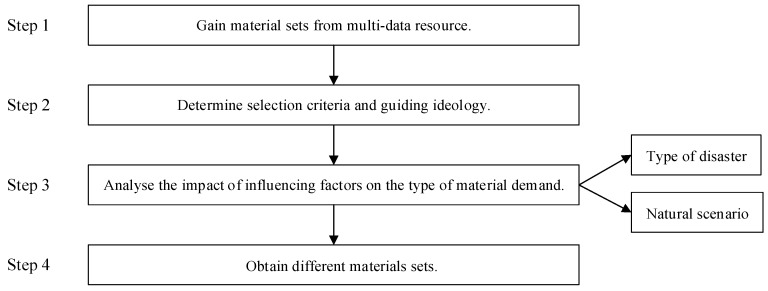
Decision workflow in the classification method.

Researchers and relief decision-makers share the knowledge that required supplies are varied, complicated, and disordered because of the occurrence of disasters, which disrupt routine/normal living conditions [64]. These complex characteristics become obstacles to HL and hinder the improvement of logistics efficiency. Scanning and sorting relief supplies are essential to ensure the effective implementation of disaster relief operation. The optimal solution is to create a material catalogue and apply it to the real world. This solution can improve the efficiency of HL through logistics means and modern supply chain theory. Figure 3 provides the steps to create this catalogue.

Step 1: Gather several sets of supplies from multiple data sources

This study gets several sets of supplies from domestic and international data sources.

Step 2: Establish the selection criteria and principles

How do we select the supplies that meet the basic demands of the victims? Material selection criteria and principles are vital because they diametrically determine the relief supplies that safeguard life and meet the needs of daily life in the event of a disaster. This study defines the selection criteria and guiding ideology of material screening as follows:

- Selected supplies should be within the categories of relief supplies.
- Generic and versatile supplies are considered.
- High-demand supplies are considered.
- Selected supplies should be related to survival and living needs.
- Available, comprehensive and convenient items should be prioritized for each type of material.

On the basis of the above criteria and principles, the four main categories of supplies are defined as survival, living, health, and rarely special.

Step 3: Analyze the effect of influencing factors on the contents of material demand

Meeting the basic demands of the victims should be considered in the material classification process to improve the rationality of the classification and the efficiency of the implementation of HL. This study considers two categories of factors that determine material classification: disaster types and natural factors [68].

##### Disaster Type $T_{j}$

Relief demand arises shortly after the occurrence of a disaster. Varying disasters lead to various demands. Although a certain overlap exists between the relief demands caused by disasters, the differences between them are significant and, therefore, increasingly noteworthy.

Discovering the difference in relief demands is worthwhile but challenging because of the different natures of disasters, such as natural hazards and human-made disasters. Risk vulnerability and disaster profiles vary according to country. For example, hurricanes, tornadoes, and floods are a few examples of frequent disasters in the United States [69]. By contrast, typhoons, floods, earthquakes, and drought are the major natural hazards in China. We only consider the disaster types that will trigger large-scale supply support and HL for the purpose of this study. The set of the selected disaster types is expressed as $T=(T_{1},T_{2},\cdots ,T_{j},\cdots T_{J})$.

Disasters bring various damages to the victims. Consequently, the demand for survival and living supplies varies according to disaster type. The demand for medical supplies widely varies because the level of injury is susceptible to the disaster type. This study considers four categories of relief demands: survival, living, medical, and special supplies. Specifically, it analyzes the differences in demand contributed by each disaster type $T_{j}$ to distinguish the effect of disaster type on these demands. Disaster type $T_{j}$ determines the differentiated supplies $M_{T_{j}}$.

##### Natural Scenario $N_{l}$

Changes in the natural environment brought about by the occurrence of disasters may lead to varying relief needs. People dealing with disasters are mainly concerned with seasonal and climate information. People often refer to “season” to generalize clothing and shelter changes or use “climate” to perceive differences amongst regions. This study introduced a precise index, namely apparent temperature, to combine different natural scenarios for determining the effect of natural factors on the type of material demand. The material demand affected by temperature should be determined, and natural scenarios combined with natural factors should be defined. $N_{l}$ represents a natural scenario combined with natural factors. For example, this study divided daily apparent temperature into two states, average night-time and average daytime temperature, and 20 °C was defined as a watershed [70,71,72]. Both temperatures have two states: H (>20 °C) and L (≤20 °C) values. These temperature states can be combined as follows on the basis of the natural law: $N_{1}$—H daytime temperature and H night temperature, $N_{2}$—H daytime temperature and L night temperature, and $N_{3}$—L daytime temperature and L night temperature. Each type of disaster corresponds to one of the three states. In each type of disaster $T_{j}$, the supplies required in certain natural states $N_{l}$ within the range of supplies affected by natural factors are denoted as $M_{{\hat{T}}_{j}N_{l}}$. Moreover, in each type of disaster $T_{j}$, supplies affected by temperature are denoted as $M_{{\hat{T}}_{j}N}$, $M_{{\hat{T}}_{j}N}=M_{{\hat{T}}_{j}N_{1}}\cup \cdots \cup M_{{\hat{T}}_{j}N_{l}}\cup \cdots \cup M_{{\hat{T}}_{j}N_{L}}$.

Step 4: Obtain the supplies catalogue for multiple disasters

In a certain type $T_{j}$ of disaster, the supplies that are unaffected by the natural factors are termed $M_{T_{j}}$. Certain supplies are affected by state $N_{l},(l=1,\cdots ,L)$, namely $M_{{\hat{T}}_{j}N_{l}}$, for a certain type $T_{j}$ of disaster. All required supplies are called $M_{T_{j}N_{l}}$ in a certain natural state $N_{l}$·$M_{T_{j}N_{l}}=M_{T_{j}}+M_{{\hat{T}}_{j}N_{l}}$ for a certain type $T_{j}$ of disaster. In a certain type $T_{j}$ of disaster, all supplies required are $M_{T_{j}N}$, which are in natural state $M_{T_{j}N}=M_{T_{j}}+M_{{\hat{T}}_{j}N}$.

We developed a material catalogue $M_{TN}$ with an application environment by using the material classification methods. Supplies required for all types of disasters studied in this study are a universal set $M_{TN}$, $M_{TN}=M_{T_{1}N}\cup \cdots \cup M_{T_{j}N}\cup \cdots \cup M_{T_{J}N}$. The material universal set $M_{TN}$ is expressed as follows: $M_{TN}=({\left(M_{TN}\right)}_{1},{\left(M_{TN}\right)}_{2},\cdots ,{\left(M_{TN}\right)}_{i},\cdots {\left(M_{TN}\right)}_{I})$.

#### 3.3.2. Calculation Process

**Figure 4:**
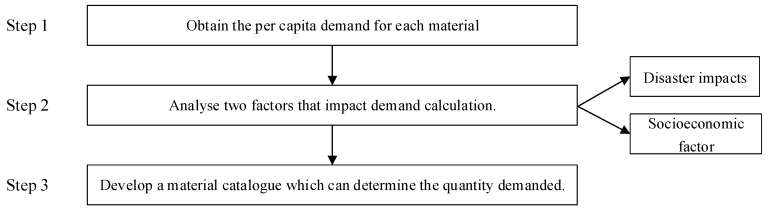
Decision workflow for the calculation method.

The former process has addressed the material classification problem of relief supplies and set up a material catalogue for dealing with disaster types and natural conditions. However, the quantity problem remains unsolved. In this subsection, we obtain the precise quantity of the demanded supplies by using a demand calculation method. We have defined two influencing factors, namely disaster impact and socioeconomic factor. Disaster impact refers to the number of victims who need relief supplies caused by various complex factors. Socioeconomic factor is a coefficient to adjust the actual supply due to economy. Figure 4 shows the steps for calculating the demand quantity of supplies.

Step 1: Obtain the per capita demand for supplies

In disaster relief operation, the minimum standard of per capita demand should be satisfied. We define the per capita demand standard in this study by referencing the guidelines of the Sphere Project and WHO standards [34,73].

Step 2: Analyze the impact factors of demand quantity

##### Disaster Impact $R_{s}$

The demand quantity of relief material is largely determined by the degree of impact of the disaster. A significant impact causes harm and loss, thereby leading to relief demand. Countries may use varying indicators to measure “disaster impact”. However, “disaster impact” is divided into different degrees, namely $\left\{R_{1},\cdots ,R_{s},\cdots ,R_{S}\right\}$. In China, this indicator refers to disaster relief emergency response levels, where $R_{1}$ and $R_{2}$ respectively represent level I and II response plans. These impact indicators can be converted into the number of victims who need relief supplies. The value for $R_{s}$ represents the number of victims who need relief supplies. In this manner, the effect of disasters and material demand are measurable quantitatively instead of qualitatively. The definition of $R_{s}$ and its value need to be analyzed according to the actual situation of a certain country or region.

##### Socioeconomic Factor $E_{c}$

Socioeconomic level is an important factor that cannot be ignored. The socioeconomic level of each country differs. The level of economic development amongst regions within a country will be different, which is closely related to the national or local disaster relief capacity without regard for international assistance. Disaster relief ability may be inadequate or exceeded due to the gap between the rich and the poor. This factor is known as socioeconomic factor $E_{c}$. The disaster relief ability as a result of socioeconomic factor $E_{c}$ can be viewed from two aspects. On the one hand, the per capita demand for the victims varies. On the other hand, per capita demand is fixed, and the number of relief population of the operation is determined in an appropriate range. This finding does not mean that the actual relief population is reduced, which would not be a moral perspective to take. The operation plans to relieve additional people can predict one’s ability to ensure supplies without reducing the per capita standard under the premise.

In the first aspect, $E_{c}$ determines whether the per capita demand for the victims is different. Specifically, the product of standard per capita demand and socioeconomic factor $E_{c}$ represents the change in per capita demand. The basic living needs of the people are different in countries with varying levels of socioeconomic development. In countries with a high socioeconomic level, providing the victims improved protection and a high standard of per capita demand is possible. By contrast, the average standard of living may not be guaranteed, and the standard of per capita demand may be reduced in countries with low social and economic levels. Thus, the international humanitarian aid per capita demand criterion has become a basic standard. The socioeconomic level of development has become a coefficient that takes 1 as the standard and may be higher or lower than 1. However, the following problem arises: How should we choose if the quantity demand of each material is an interval value in a certain $R_{s}$?

**Figure 5:**
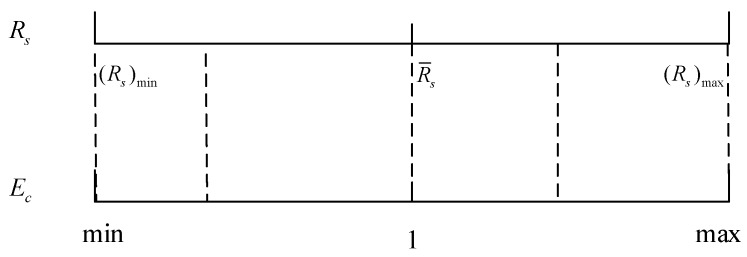
Correlation between socioeconomic coefficient $E_{c}$ and relief population $R_{s}$.

In the second aspect, the relief material population of the operation plan determined/caused by the disaster impact $R_{s}$ is an interval value. $E_{c}$ is used to determine/measure an exact/constant value in this interval/range. Specifically, the value chosen within the interval is high when the socioeconomic coefficient $E_{c}$ is also high. This condition entails stocking extra supplies. The value chosen within the interval is small when the socioeconomic coefficient $E_{c}$ is also small, thereby indicating stocking less supplies. The mean value in this interval corresponds to a value of $E_{c}=1$. If so, we obtain an exact/constant value in any manner rather than an interval value, which plays an important role in the implementation of HL and the pre-storage of supplies. Figure 5 shows the correspondence between socioeconomic coefficient $E_{c}$ and relief population $R_{s}$.

Given a value of $E_{c}$ in an appropriate range, a specific value of $R_{s}$ exists in the corresponding interval. Socioeconomic coefficient $E_{c}$ can be expressed by GDP and the Gini and Engel coefficients, which are entirely dependent on the country or region. The range of socioeconomic coefficient $E_{c}$ is $E_{c}=[\frac{{\left(R_{s}\right)}_{\min}}{{\bar{R}}_{s}},\frac{{\left(R_{s}\right)}_{\max}}{{\bar{R}}_{s}}]$.

Step 3: Develop a material catalogue that can determine the demanded quantity

We obtain a material set $M_{TN}$ through material classification. The set contains four categories/classes of supplies: survival, living, health, and special supplies.

Per capita demand $P_{{\left(M_{TN}\right)}_{i}}$ and the number of victims who need relief supplies are obtained through analysis of demand calculation methods in terms of survival and living supplies. The value for $R_{s}$ is equal to the number of victims that need relief supplies. $E_{c}$ refers to the socioeconomic coefficient. If $R_{s}$ is an exact/constant value rather than an interval, then $Q_{{\left(M_{TN}\right)}_{i}}={\left(M_{TN}\right)}_{i}\cdot [P_{{\left(M_{TN}\right)}_{i}}\cdot R_{s}\cdot E_{c}]$. If $R_{s}$ is an interval value, then $Q_{{\left(M_{TN}\right)}_{i}}={\left(M_{TN}\right)}_{i}\cdot [P_{{\left(M_{TN}\right)}_{i}}\cdot {\bar{R}}_{s}\cdot E_{c}]$, $E_{c}=[\frac{{\left(R_{s}\right)}_{\min}}{{\bar{R}}_{s}},\frac{{\left(R_{s}\right)}_{\max}}{{\bar{R}}_{s}}]$. A material catalogue to determine the demand amount can be established on the basis of the classification set ${\left(M_{TN}\right)}_{i}$ and corresponding calculation results $Q_{{\left(M_{TN}\right)}_{i}}$.

The demand quantities for medical and special supplies should be calculated in light of the state’s rescue opinion and actual rescue experience.

## 4. Application (in China)

In this section, we conduct a case study that applies the proposed method to China’s disaster background to demonstrate the basic process. This method can be applied not only in China but also in other countries. Relief organizations can obtain reliable demand for relief supplies by following the method.

### 4.1. Classification Process

Step 1: Obtain several material sets from multiple data resources

We query material catalogues from China, the United States, and international organizations. The literature review section presents a portion of the datasets.

Step 2: Select supplies from sets according to the abovementioned ideology

Based on the disaster management background in China, different administrative systems are responsible for the reserve of different categories of supplies; thus, four categories of supplies, namely survival, living, health, and special supplies, are identified [3,17,74].

Step 3: Consider and analyze the influencing factors of material classification 

First, identify the major disaster types in China and analyze the relief demand caused by each disaster. Second, identify the relief supplies influenced by natural scenarios.

#### 4.1.1. Determine Disaster Types and Relief Needs

Historical data reported by National Disaster Reduction Center of China show that four major types of disasters require large quantities of humanitarian relief supplies in China, namely earthquakes, floods, typhoons, and drought, [65]. Thus, this study only considers these disaster types that cause a significant demand in relief supplies.

We identify the supplies that are unaffected by natural factors but are directly determined by disaster type. The relief demand for these supplies is triggered only by disasters and remains unaffected by natural factors. We call these supplies disaster-determining supplies and represent them as a material set $M_{T_{j}}$ if the disaster type is $T_{j}$. Table 2 lists the disaster-determining supplies of survival, living, and special supplies. Detailed healthcare supplies are given in Appendix A (except the demand column).

This section determines the major disaster types and vast majority of relief supplies for each disaster type in four categories.

#### 4.1.2. Analysis of Natural Factors and Affected Supplies

As described in the previous section, three temperature scenarios are identified: $N_{1}$ denotes an average daytime temperature >20 °C and night temperature >20 °C, $N_{2}$ implies an average daytime temperature >20 °C and average night temperature ≤20 °C, and $N_{3}$ represents an average daytime temperature ≤20 °C and night temperature ≤20 °C. The supplies selected in this portion are affected by natural factors and follow the guiding principles. Under disaster type $T_{j}$, supplies affected by specific scenarios $N_{l}$ are denoted as $M_{{\hat{T}}_{j}N_{l}}$. On the basis of the guiding principle, we identify the supplies affected by natural factors under various disasters, such as earthquakes, typhoons, flood, and drought. Table 3 lists the living supplies affected by the three temperature states. In disaster type $T_{j}$, supplies affected by natural factors are denoted as $M_{{\hat{T}}_{j}N}$, $M_{{\hat{T}}_{j}N}=M_{{\hat{T}}_{j}N_{1}}\cup \cdots \cup M_{{\hat{T}}_{j}N_{l}}\cup \cdots \cup M_{{\hat{T}}_{j}N_{L}}$. The analytical result shows that natural states/conditions exert the same impact on relief supplies for earthquakes, typhoons, and flood. Therefore, the affected supplies remain the same, that is, $M_{{\hat{T}}_{1}N}=M_{{\hat{T}}_{2}N}=M_{{\hat{T}}_{3}N}$. During drought, relief supplies are unaffected by natural factors.

Step 4: Obtain different material sets for each disaster type

Under disaster type $T_{j}$ and specific natural states/conditions $N_{l}$, the total required relief supplies are denoted as $M_{T_{j}N_{l}}$, $M_{T_{j}N_{l}}=M_{T_{j}}+M_{{\hat{T}}_{j}N_{l}}$. $M_{T_{j}N}$ represents the required relief supplies for disaster $T_{j}$, $M_{T_{j}N}=M_{T_{j}N_{1}}\cup \cdots \cup M_{T_{j}N_{l}}\cup \cdots \cup M_{T_{j}N_{L}}$; and $M_{TN}$ represents the supplies for the four types of disasters, namely $M_{TN}=M_{T_{1}N}\cup \cdots \cup M_{T_{j}N}\cup \cdots \cup M_{T_{J}N}$. For example, the supplies required for earthquakes are those for earthquake as cited in Table 2 and Table 3 and Appendix A (except for the demand column). Duplicate supplies were removed from Table 3. In most cases, one natural state for disaster relief exists. Therefore, the required supplies are those for earthquake, as mentioned in Table 2, Appendix A (except for the demand column) and the supplies of natural state $N_{1}$, $N_{2}$, or $N_{3}$ in Table 3. The supplies under the four types of disasters studied in this work are those mentioned in Table 2 and Table 3 and Appendix A (except for the demand column). Duplicate supplies were removed from Table 3.

The following findings were obtained according to a previous analysis.

Relief demand for earthquakes covers the living, survival, and medical supplies (e.g., equipment, commonly used and anti-epidemic drugs, etc.). Professional supplies are unnecessary needs for earthquake relief.

Relief demand for typhoons covers all living, survival, and medical supplies (e.g., equipment, commonly used and anti-epidemic drugs, etc.). The listed professional supplies are in demand.

Relief demand for flood covers all living and survival supplies and part of the medical supplies. Equipment and certain commonly used drugs are not in demand. The listed professional supplies are in demand.

Relief demand for drought only covers part of survival supplies and commonly used and anti-epidemic drugs. The living supplies, equipment, and some commonly used drugs are unnecessary.

Each type of material is also classified.

Survival and living supplies are always needed in earthquakes, typhoons, and flood. Raincoats and rain boots are needed in the case of rain during earthquakes.

Healthcare supplies cover three categories, namely medical equipment, commonly used drugs, and epidemic prevention supplies. Medical equipment is only needed in earthquake and typhoon in the case of mass disaster wounds (head, skin, nerves, tendon, fracture, chest, and abdomen and multiple injuries) caused by high rescue complexity [75]. Amongst the 11 common drugs, only 3 are needed during earthquakes and typhoons.

Special supplies, assault/rubber/speedboats and life jackets/buoys are in great demand during typhoons and floods.

### 4.2. Calculation Process

Step 1: Obtain the per capita demand for each material

We used the Sphere Handbook and WHO standards to generate per capita demand for relief supplies and combined them with China’s relief experience. Tables 6 and 7 present the per capita demand of each material for survival and living supplies. We consulted the “Health emergency response team equipment references (trial)” by the Chinese Ministry of Health in addition to the Sphere Handbook Core Standards for medical supplies. Practical experience was likewise considered.

Step 2: Analyze the factors that impact demand calculation

#### 4.2.1. Disaster Impacts

The disaster impact in China is expressed as the number of victims who need relief in different emergency response levels. In China’s disaster relief system, response is divided into four levels, which represent the degrees of disaster impact. A correspondence exists between disaster relief emergency response level and the number of victims who need relief supplies. We summarized this correspondence in Table 4 after consulting the emergency response standard in China and reviewing the disaster relief history data of China.

The values in Table 4 represent the number of victims who need relief supplies under varying response levels of disaster types. The columns indicate that for the same response level, the number of victims who need relief supplies varies according to the type of disaster. The numbers of victims in need of relief are similar for these four disasters in different relief response levels. Such findings are conducive to disaster management and relief practice. Table 5 provides the aggregated results. We obtain the demand amount for survival and living relief supplies under each response level by combining the table values with per capita demand data.

#### 4.2.2. Socioeconomic Factor

The current economic level in China has sufficient capacity to meet the per capita demand standard for international humanitarian assistance. Therefore, we set the socioeconomic coefficient to 1. The demand quantity of each material is estimated as an interval number according to the current situation in China. Given $E_{c}$ as a definite value from an appropriate range, a corresponding $R_{s}$ is derived from the interval.

Step 3: Develop a material catalogue that can determine the demand amount

The demand quantity for survival and living supplies can be calculated using the formula $Q_{{\left(M_{TN}\right)}_{i}}={\left(M_{TN}\right)}_{i}\cdot [P_{{\left(M_{TN}\right)}_{i}}\cdot {\bar{R}}_{s}\cdot E_{c}]$. We can create a quantitative material catalogue to determine the demand amount by combining the calculated numbers with material classification sets ${\left(M_{TN}\right)}_{i}$. Table 6 and Table 7 provide the catalogues for survival and living supplies, respectively.

The demand calculation process for medical and special supplies should follow the judgment and actual experience of the Chinese rescue team. Appendix A provides the material catalogues for medical and special supplies.

Table 6 lists the total demand for each survival material under each response level. The contents in Table 6 are briefly explained below. The first column of the table represents the type of disaster. The second column represents the contents of supplies. Finally, the third column provides the per capita demand for each material. Every row in the table, except the header, represents the disaster types, which are applicable to each material and the per capita demand and quantities demanded under varying impacts of disaster. Similar to material purified water, this material is required for four types of disasters. The per capita demand is 15 l per person per day. The quantities demanded are 65,625, 42,000, 23,625, and 10,500 t under the I, II, III, and IV response levels, respectively.

Table 7 displays the living material category, per capita demand, and total demand for goods under various degrees. Supplies that are unaffected by natural states and those needed in any state of $N_{1}$, $N_{2}$, and $N_{3}$ are needed in terms of living supplies required during earthquake/typhoon/flood. Moreover, supplies in states $N_{1}$, $N_{2}$, and $N_{3}$ have a certain redundancy, but they are highly conducive to decision-making.

The contents of Table 7 are briefly explained below. The first, second, third, and fourth columns represent the type of disaster, a detailed classification caused by natural factors, contents of supplies, and per capita demand for the corresponding material, respectively. Except for the header, each row represents the type of disaster and natural state applicable to each material, per capita demand and quantities demanded under the impact of disaster. Similar to material tents, this material is required for three types of disasters and is unaffected by the natural state. The per capita demand is one-fourth. The quantities demanded are 156,250, 100,000, 57,500, and 26,667 under the I, II, III, and IV response levels, respectively. For example, the per capita demand for material tents (12 m^2^) is one-fourth. If the Chinese government starts at the level I relief response, then the demand for this material is 156,250.

Relief supplies, such as tent and color bar cloth, sleeping bag, quilt and blanket, cotton coat, and cold protective clothing (down jacket and warm clothing), can be substituted. Decision-makers can select substitutable supplies with proper proportion for the same or similar product function.

In summary, this study mainly considers survival, living, and healthcare supplies which victims need and policy-makers consider. It conducts an in-depth research on the guiding ideology of material selection with reference to national or international guidance documents and standards. A material catalogue is created on the basis of the classification method. We derive material quantity demand lists by analyzing the actual situation in China. These results suggest that we can also obtain a list on relief material quantity demanded under other geographical features as long as we rely on our methods and basic principles in combination with the actual application background of other countries.

## 5. Numerical Case

We assume that the government plans to build a warehouse in Anhui, China, to deal with disasters. This warehouse aims to deal with level II flood disaster. The decision-makers want to know the supplies that they should reserve and the quantities. Decision-makers can refer to survival supply types in Table 2 and Table 3 (and remove duplicate supplies) and living and special supplies in Table 2 for flood disaster. Medical supply data can be derived from the Appendix for flood disaster. Decision-makers can obtain the demand quantity for each material needed for flood disaster in level II.

The government will launch a level II response when an earthquake occurs in Anhui, China. Decision-makers want to know the supplies that they should deliver to disaster areas and the amounts. First, decision-makers are aware that the disaster type is an earthquake. These individuals should then explore the natural states in the disaster area. We assume that daytime temperature is 23 °C, while the night temperature is 15 °C in the affected area. The natural states in the affected area are classified under $N_{2}$ on the basis of Table 3. The decision-makers can obtain a part of survival material types in Table 2 and another part under natural state $N_{2}$ in Table 3 for earthquake disasters. They can gain living and special supplies in Table 2 for earthquake disaster and obtain medical supplies from the Appendix for earthquake. Finally, decision-makers can obtain the demand quantity for each material needed for earthquake disasters in level II.

## 6. Conclusions

Determining the demand item and quantity contributes to improve warehousing efficiency of relief supplies and speed up relief response. However, previous studies in supply classification or demand prediction did not clarify the types of supply required and quantities demanded for each item. No studies have been done on the combination of supply classification and demand calculation that can obtain the checklist of demand supply with quantity for decision-makers in post-disaster relief. A method for solving the gap is proposed. The method has practical significance and provides valuable suggestions and guidelines for disaster management staff. Instead of two separate studies, this study integrates material classification and demand calculation into connected processes, in which the results of supply classification are passed to demand calculation process. The relief demand comprises the contents and quantities of demand, respectively. We first classify the relief supplies according to disaster type and natural factors and then establish material catalogues applicable to various disaster relief scenarios. Thereafter, the demand quantity is calculated on the basis of the disaster impact and socioeconomic factors. We demonstrate the usefulness of our method on a case study based on the disaster situation in China. Results show that the developed method can assist the disaster management staff in quickly considering relief needs, which have yet to be solved by other methods thus far.

This study is not limited to one country or region. The developed method provides a general rule for material classification and demand calculation. This rule will offer conducive and executable suggestions to the disaster management staff. Enhancing the disaster management level and emergency support capability are of great practical significance.

Material classification and demand calculation are long-term research topics in the HL field. However, the present study is an exploratory research on this topic. Generalizing a perfect result from a single study is an impractical approach. The extendibility of this study is worthy of further research to validate its results.

This study has several limitations despite its contribution. A significant limitation is the lack of a detailed discussion on medical and specific supplies owing to insufficient professional knowledge. Classification and calculation results are missing or incomplete, especially drugs. The per capita demand standards adopted by the study are unsuitable worldwide. This condition indicates that these criteria are not the only ones available or immutable. Once such standards are set and enforced in a region, the demands become certain. If a slight difference exists in the standard setting of per capita demand in some regions, then the results on demand are uncertain and different. However, this uncertainty is foreseen in advance and already estimated. Finally, a common data pool should be established for allowing researchers to obtain disaster-related data and their respective effects on beneficiaries.

We follow the steps of the classification method. Taking the Chinese disaster background as a case study, we obtain the medical supply catalogue and its application environment. Medical supplies are divided into three classes: equipment, commonly used drugs, and epidemic prevention class. With regard to demand, the amount of victims is an improper indicator. The number of injured persons will be limited, but the severities of injuries will also differ amongst affected persons. The quantity demanded for medical supplies is derived from the actual rescue experience in China and adheres to the “Health emergency team equipment reference catalogue (Trial)” promulgated by the Ministry of Health P.R. China. The number of medical equipment in Appendix A can provide the 40 healthcare workers 30 beds and 2 operating tables, help them carry out emergency rescue 7–10 days, and care for 200–300 sick and wounded daily (24 h). The demand for commonly used medicines and anti-epidemic drugs should be determined by professionals because these supplies are highly risky. The types of drugs are also complex and diverse. Such circumstance leads to a great difficulty for the storage, transportation, and management of drugs. The drug storage category can also be reduced. Each medicine system stores a large number of single varieties. The demands for special supplies are also determined by professionals.

## Figures and Tables

**Table 1:** Meaning and definition of symbols.

Symbol	Meaning
$M_{T_{1}}$	Supplies directly determined by disaster type $T_{1}$ and unaffected by natural environmental factors
$M_{{\hat{T}}_{1}N_{1}}$	Supplies required in natural states/conditions $N_{1}$ in the range of supplies affected by natural environmental factors in disaster type $T_{1}$
$M_{{\hat{T}}_{1}N}$	Supplies affected by natural environmental factors in disaster type $T_{1}$
$M_{T_{1}N_{1}}$	Supplies required in natural states/conditions $N_{1}$ of disaster type $T_{1}$
$M_{T_{1}N}$	Supplies required in disaster type $T_{1}$
$M_{T_{j}}$	Supplies directly determined by disaster type $T_{j}$ and unaffected by natural environmental factors
$M_{{\hat{T}}_{j}N_{l}}$	Supplies required in natural states/conditions $N_{l}$ in the range of supplies affected by the natural environmental factors in disaster type $T_{j}$
$M_{{\hat{T}}_{j}N}$	Supplies affected by the natural environmental factors in disaster type $T_{j}$
$M_{T_{j}N_{l}}$	Supplies required in natural states/conditions $N_{l}$ of disaster type $T_{j}$
$M_{T_{j}N}$	Supplies required in disaster type $T_{j}$
$M_{TN}$	Supplies required for all types of disasters studied in this article (universal set)

**Table 2:** Survival, living, and special supplies directly determined by disaster type.

Disaster Type	Material Type	Material Name
Earthquake/Typhoon/Flood	Living Supplies	Tent (12 m^2^)
		Color Bar Cloth
		Folding Bed/Inflatable Pad
		Folding Tables and Chairs
		Hand-cranked/Flash/Emergency/Waterproof Light
		Simple Toilet
		Trash Cans
		Garbage Bag (Roll)
		Disinfectant (250 mL)
		Cleaning Supplies (200 g Laundry Soap and Body Soap)
		Personal Hygiene Products
		Raincoat
		Rain Boots
Earthquake/Typhoon/Flood/Drought	Survival Supplies	Purified Water
		Flour/Rice
		Edible Oil
		Edible Salt
		Vitamin Complex Tablets
Earthquake/Typhoon/Flood		Water Container 10–20 L
		Stainless Steel Pots (Cookware and Tableware)
Typhoon/Flood	Special Supplies	Assault/Rubber/Speedboat
		Life Jackets/Buoy

**Table 3:** Supplies required in each natural state for each disaster type in the range of supplies affected by natural factors.

Disaster Type	Material Type	Natural States/Conditions	Material Name
Earthquake/Typhoon/Flood	Living Supplies	$N_{1}$	Towel Quilt
			Mosquito Net
			Sportswear
		$N_{2}$	Quilt/Blanket/Sleeping Bag
			Sportswear
		$N_{3}$	Sleeping Bag
			Quilt/Blanket
			Cold Protective Clothing (Down Jacket, Warm Clothing, and Cotton Coat)
			Cotton socks

**Table 4:** Correspondence between the response level and the number of victims who need relief supplies during disasters.

Response Level	Disaster Type		
	Earthquake	Typhoon	Flood
I	>500.000	>500.000	>600.000
II	300.000–500.000	300.000–500.000	300.000–600.000
III	120.000–300.000	160.000–300.000	150.000–300.000
IV	<120.000	30.000–160.000	15.000–150.000

**Table 5:** Synthetic correspondence between the disaster response level and the number of victims who need relief supplies.

Response Level	Population that Needs Relief Supplies
I	>500.000
II	300.000–500.000
III	150.000–300.000
IV	<150.000

**Table 6:** Survival material catalogue to determine the demand amount used in Chinese HL (humanitarian logistics) practice.

Disaster Type	Material Name	Per Capita Demand $E_{c}=1$	Response Level			
			I	II	III	IV
Earthquake/Typhoon/Flood/Drought	Purified Water	15 L per person per day (rescue days = 7)	65.625 *t*	42.000 *t*	23.625 *t*	10.500 *t*
	Flour/Rice	0.6 kg per person per day (rescue days = 7)	2625 *t*	1680 *t*	945 *t*	420 *t*
	Edible Oil	25 g per person per day (rescue days = 7)	109.375 *t*	70 *t*	39.375 *t*	17.5
	Edible Salt	6 g per person per day (rescue days = 7)	26.25 *t*	16.8 *t*	9.45	4.2
	Vitamin Complex Tablets	One bale per tent, 1/4	156.250	100.000	57.500	26.667
Earthquake/Typhoon/Flood	Water Container 10–20 L	1/2	312.500	200.000	112.500	50.000
	Stainless Steel Pots (Cookware, Tableware)	1/4	156.250	100.000	57.500	26.667

**Table 7:** Living material catalogue to determine the demand amount used in Chinese HL practice.

Disaster Type	Natural States	Material Name	Per Capita Demand $E_{c}=1$	Response Level			
				I	II	III	IV
**Earthquake/Typhoon/Flood**		Tent (12 m^2^)	1/4	156.250	100.000	57.500	26.667
		Color Bar Cloth	2.5 kg	1562.5	1000 *t*	562.5 *t*	250 *t*
		Folding Bed/Inflatable Pad	One per Person	625.000	400.000	225.000	100.000
		Folding Tables and Chairs	One per Tent, 1/4	156.250	100.000	57.500	26.667
		Hand-cranked/Flash/Emergency/Waterproof Lights	1/4	156.250	100.000	57.500	26.667
		Simple Toilet	1/50	12.500	8000	4500	2000
		Trash Cans	1/100	6250	4000	2250	1000
		Garbage Bag (Roll)	1/4	156.250	100.000	57.500	26.667
		Disinfectant (250 mL)	Per Person per Month	625.000	400.000	225.000	100.000
		Cleaning Supplies (200 g Laundry Soap and Body Soap)	One per person per month	625.000	400.000	225.000	100.000
		Personal Hygiene Products	Per Person per Month	625.000	400.000	225.000	100.000
		Raincoat	1/100	6250	4000	2250	1000
		Rain Boots	1/100	6250	4000	2250	1000
	$N_{1}$	Towel Quilt	1	625.000	400.000	225.000	100.000
		Mosquito Net	1	625.000	400.000	225.000	100.000
		Sportswear	1	625.000	400.000	225.000	100.000
	$N_{2}$	Sleeping Bag	1	625.000	400.000	225.000	100.000
		Quilt/Blanket	1	625.000	400.000	225.000	100.000
		Sportswear	1	625.000	400.000	225.000	100.000
	$N_{3}$	Sleeping Bag	1	625.000	400.000	225.000	100.000
		Quilt/Blanket	1	625.000	400.000	225.000	100.000
		Cold Protective Clothing (Down Jacket, Warm Clothing and Cotton Coat)	1	625.000	400.000	225.000	100.000
		Cotton Socks	One Person; Two Pairs	1.250.000	800.000	450.000	200.000

## Appendix A

**Table A1:** Health and special supply catalogue with demand.

Disaster Type	Supply Class		Material Name	Demand
Earthquake/Typhoon	Equipment	Carry Equipment	First Aid Kit	5
			Resuscitation (Anti-shock) Backpack	5
			Primary Debridement Backpack	5
			Infusion of Drugs for the Backpack	5
			Handling Backpack	5
		First Aid Equipment	Defibrillator	1
			Infusion Pump	10
			Neck Brace (Head Holder)	20
		Surgical Equipment	Debridement Suture Kit	30
			Dressing Bag	30
			Urethral Catheterization Bag	30
			Tracheal Incision Package	30
			Intravenous Incision Package	4
			Deep Vein Puncture Bag	4
			Orthopedic Equipment Package	10
			Chest Equipment Package	3
			Brain Surgery Equipment Package	3
			Exploratory Laparotomy Bag	4
			Gynecologic Surgical Exploration Kit	2
			Vascular stapler	2
			High-frequency Electric Knife	2
			Surgical Bed (Portable)	2
			Surgical Lights (Portable)	2
			Portable Instrument Platform	2
			Anesthesia Machine (Portable)	2
			Breathing Machine (Electric)	2
			Suction Device (Electric)	5
			Surgical Suction Machine	2
			Field Hand Washing Device	2
			Monitor (Multiple Parameters)	6
			Autologous Blood Transfusion Device	1
			Surgical Equipment Supply Box	1
		Special Equipment	ECG Machine	1
			B Ultrasonic (Portable)	1
			X-ray Machine (Portable)	1
			Washing Machine (Ming room)	1
			Field Clinic Bed (Folding)	2
		Disinfection Supply Equipment	Autoclave Sterilizer	2
			Multi-person Oxygenator	2
			Small Medical Pure Water Device	1
			Freezer (Chest of Blood)	2
			Oxygen Cylinders (40 L)	5
		Inspection Equipment	Medical Refrigerator (50 L)	2
			Microscope	1
			Centrifuge	1
			Coagulation Analyzer	1
			Biochemical Analyzer (Dry)	1
			Blood Cell Count Instrument	1
			Blood Type Test Equipment	1
			Inspection Equipment Supply Box	1
		Facial Feature Equipment	Frontier Inspection Equipment Box	1
		Epidemic Prevention Equipment	Check the Water Seized Drug Box	1
			Motorized Sprayer	1
			Manual Sprayer	1
		Motorized Health Equipment	Ambulance	2
			Medical Box Group	1
			Combined Medical Tent Unit	1
		Other Equipment	Stretcher (Folding, Hard and Shovel)	15
			Hospital Bed (Folding)	30
			Nursing Equipment Supply Box	1
			Medical Equipment Repair Box	2
			Generator	A certain number
			Inspection Mark	A certain number
Earthquake/Typhoon/Flood/Drought	Commonly Used Drugs		Antibiotic	Decided by professionals
			Antipyretic Analgesic Drugs	
			Water, Electrolyte Balance Drugs	
			Respiratory Medication	
			Digestive System Medication	
			Disinfectant	
			Antiparasitic Drugs	
			Skin Disease Medication	
Earthquake/typhoon/			Blood System Drugs	
			Sedative and Anti-allergy Drugs	
			Anesthesia and Cardiovascular System Rescue Drugs	
Earthquake/Typhoon/Flood/Drought	Epidemic Prevention Class		Calcium Hypochlorite Disinfection Tablets/Bleaching Powder/Effervescent Tablets	
Earthquake/Drought	Special Supplies		Nothing	
Typhoon/Flood			Assault/Rubber/Speed Boat	
			Life Jackets/Buoy	

The demand expressed in the fourth column can meet the needs of a medical team (40 people) carrying out emergency relief work (7 days). In addition, 200–300 individuals are wounded every 24 h.
